# Relationship of Buckling and Knee Injury to Pain Exacerbation in Knee Osteoarthritis: A Web-Based Case-Crossover Study

**DOI:** 10.2196/ijmr.5452

**Published:** 2016-06-24

**Authors:** Isabelle Zobel, Tahereh Erfani, Kim L Bennell, Joanna Makovey, Ben Metcalf, Jian Sheng Chen, Lyn March, Yuqing Zhang, Felix Eckstein, David J Hunter

**Affiliations:** ^1^Institute of Anatomy, Paracelsus Medical University SalzburgSalzburgAustria; ^2^Sydney Medical School, University of SydneySydneyAustralia; ^3^Centre for Health, Exercise and Sports Medicine, Department of Physiotherapy, University of MelbourneMelbourneAustralia; ^4^Institute of Bone and Joint Research, Kolling Institute, University of Sydney, Sydney, NSW AustraliaSt LeonardsAustralia; ^5^Institute of Bone and Joint Research, Kolling Institute, University of Sydney, and Rheumatology Department, Royal North Shore Hospital, Sydney, NSW AustraliaSydneyAustralia; ^6^Boston University School of Medicine, Clinical Epidemiology Research and Training Unit, Boston, MABoston, MAUnited States; ^7^Institute of Bone and Joint Research, Kolling Institute, University of Sydney, and Rheumatology Department, Royal North Shore Hospital, Sydney, NSW AustraliaSt LeonardsAustralia

**Keywords:** knee osteoarthritis, injury, buckling pain exacerbation, case-crossover study, web-based

## Abstract

**Background:**

Knee osteoarthritis (OA) is one of the most frequent causes of limited mobility and diminished quality of life. Pain is the main symptom that drives individuals with knee OA to seek medical care and a recognized antecedent to disability and eventually joint replacement. Evidence shows that patients with symptomatic OA experience fluctuations in pain severity. Mechanical insults to the knee such as injury and buckling may contribute to pain exacerbation.

**Objective:**

Our objective was to examine whether knee injury and buckling (giving way) are triggers for exacerbation of pain in persons with symptomatic knee OA.

**Methods:**

We conducted a case-crossover study, a novel methodology in which participants with symptomatic radiographic knee OA who have had knee pain exacerbations were used as their own control (self-matched design), with all data collected via the Internet. Participants were asked to log-on to the study website and complete an online questionnaire at baseline and then at regular 10-day intervals for 3 months (control periods)—a total of 10 questionnaires. They were also instructed to go to the website and complete pain exacerbation questionnaires when they experienced an isolated incident of knee pain exacerbation (case periods). A pain exacerbation “case” period was defined as an increase of ≥2 compared to baseline. At each contact the pain exacerbation was designated a case period, and at all other regular 10-day contacts (control periods) participants were asked about knee injuries during the previous 7 days and knee buckling during the previous 2 days. The relationship of knee injury and buckling to the risk of pain exacerbation was examined using conditional logistic regression models.

**Results:**

The analysis included 157 participants (66% women, mean age: 62 years, mean BMI: 29.5 kg/m^2^). Sustaining a knee injury was associated with experiencing a pain exacerbation (odds ratio [OR] 10.2, 95% CI 5.4, 19.3) compared with no injury. Knee buckling was associated with experiencing a pain exacerbation (OR 4.0, 95% CI 2.6, 6.2) compared with no buckling and the association increased with a greater number of buckling events (for ≥ 6 buckling events, OR 20.1, 95% CI 3.7, 110).

**Conclusions:**

Knee injury and buckling are associated with knee pain exacerbation. Reducing the likelihood of these mechanical events through avoidance of particular activities and/or appropriate rehabilitation programs may decrease the risk of pain exacerbation.

## Introduction

Osteoarthritis (OA) is a leading cause of musculoskeletal pain and disability where the knee is commonly affected [1]. Pain is dominant, becoming persistent and more limiting with disease progression, leading to disability, reduced quality of life, and often joint replacement [2]. Pain results from a complex interaction between structural joint changes [3], physical impairments [4], and psychosocial factors [4]. Evidence has shown that patients with symptomatic OA experience fluctuations of relatively short durations in pain severity [2,5,6]. These intense and intermittent pain fluctuations are called pain exacerbations or pain flares [7]. Pain flares are often exacerbated during activities and are relieved with rest, although later in the disease course, pain can also occur at night and during rest [2,7]. If we are to impact the experience of pain, particularly intermittent pain having a greater impact on quality of life [5], then identification of factors predisposing to fluctuations in pain severity is crucial.

Mechanical insults to the knee such as injury and buckling may contribute to pain exacerbation. The latter is defined as giving way during weight bearing activities [8]. The buckling event can be caused by pain, knee instability, or insufficient muscle strength, and furthermore, may lead to injurious falls [8].A cross-sectional study suggested that almost 12% of participants with knee OA had at least one event of knee buckling during the last 3 months and that buckling was associated with functional loss and limitation of daily activities [8]. The study of Nguyen et al [9] reported that within 3 months study period among those who suffered knee buckling, the overwhelming majority had two or more episodes of knee buckling during that time. For these participants, 74% reported 2-5 buckling episodes, 17% reported 6-10 episodes, and 9% reported 11 or more episodes [9]. Knee buckling and especially sensations of knee instability without buckling were found to be associated with fear of falling, poor balance confidence, activity limitations, and poor physical function [9]. To date however, the role of knee injury and buckling in predisposing to exacerbations of pain in persons with knee OA remains unclear. Thus we aimed to investigate this relationship using an Web-based case-crossover study.

## Methods

### Study Design

We conducted a Web-based case-crossover study to investigate the relationship between knee buckling and/or knee injury and OA knee pain exacerbation as described in our recent protocol paper [10]. This design is ideally suited to assess the effects of transient and intermittent exposures (triggers) on recurrent acute events (such as pain flares). The case-crossover study uses each participant as his or her own control and compares the frequency of exposure to a suspected precipitating factor (eg, knee buckling and/or knee injury) from an acute episode onset (case period, ie, a knee OA pain exacerbation in this study) to that during control periods. Self-matching of each participant minimizes the bias in control selection and removes the confounding effects of factors that are constant over time [11,12]. The case-crossover study design has been successfully utilized to evaluate associations between transient exposures and the onset of acute events [13-15]. A case-crossover study design can also be used to evaluate risk factors for recurrent acute events such as multiple pain exacerbations during the study follow-up period [13]. A participant can contribute both case and control periods multiple times during the study follow up.

### Study Population

Participants were recruited from existing databases at the Royal North Shore Hospital of Sydney and the University of Melbourne that included people diagnosed with symptomatic radiographic knee OA who had agreed to be contacted for future studies. Another source of recruitment was volunteers responding to advertisements placed in the general community and in health care settings.

A set of prescreening questions was used to identify eligible participants. All participants had to undergo knee X-rays. The inclusion criteria for participation were: 1) age above 40 years; 2) pain on most days (≥15 days) of the previous month; 3) fluctuations in pain level; 4) at least one knee that met the American College of Rheumatology criteria of OA (knee pain, stiffness, or aching) [16]; 5) tibiofemoral Kellgren and Lawrence Grade (KLG)≥ 2 [17] or patellofemoral [18] OA documented on a radiograph; 6) no plan to have a total knee replacement in the symptomatic knee in the next 6 months; 7) no history of OA secondary to inflammatory joint disease, osteonecrosis, Paget’s disease, etc; 8) have an active email account and access to the Web; and 9) have good understanding of spoken and written English. This study was approved by the Human Ethics Committee of the University of Sydney and the University of Melbourne and all participants provided informed consent. Participants were screened and enrolled via the website. The study participants were followed for 3 months and were asked to complete the online questionnaires at the commencement of the study (baseline) and then at regular 10-day intervals.

### Ascertainment of Knee Pain Exacerbation

**Figure 1:**
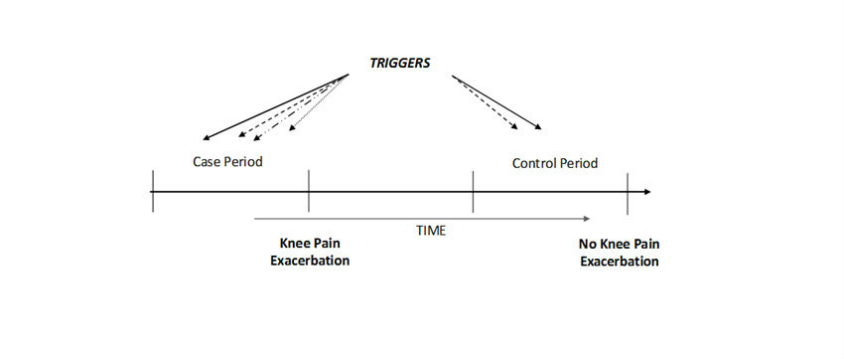
Case-crossover study design and timing of exposure measurements in relation to knee osteoarthritis pain exacerbation.

At the commencement of the study, we asked participants to rate the severity of their current knee pain using a 0-10 numeric rating scale (NRS) with zero being “no pain” and 10 being “extreme pain.” Each questionnaire was preceded by a clear description of how to complete it. Participants were instructed to log-on to the study website and complete the questionnaires when they experienced an increase in their pain level (a disabling increase in their knee symptoms that lasted for longer than 8 hours without settling) (Figure 1). The online study website then automatically calculated and determined whether the current knee pain was defined as a “pain exacerbation” by comparing it with their baseline pain level measured as the mildest level of their background pain on the NRS scale (defined as an increase of ≥ 2 on a 0-10 numerical rating scale from baseline [19]. Participants were prompted to complete the online questionnaires by means of automated reminder emails. If participants did not meet this threshold and were not due to complete their regular 10 day questionnaire, no further information was collected. If two pain exacerbations occurred within 7 days, then the subsequent pain exacerbation was excluded from the analysis. The content of the questionnaires for the case and regular 10-day contact visits was the same, with both ascertaining the frequency of knee buckling and knee injuries.

### Ascertainment of Knee Injury and Buckling

Knee buckling episodes were assessed by recall in the first 2 days and have been found to be common in patients with knee OA. As knee injuries are likely to be less frequent than buckling and as we also wanted to capture minor injuries (which may have a longer interval from injury to pain exacerbation), our interval of assessment of the occurrence of knee injury was extended to the first 7 days.

The participants were asked whether they experienced knee buckling, defined as giving way [8], during the previous 2 days; how often the knee buckled during that period of time; whether they fell during that event; and what they were doing when the knee buckled? These self-reported instruments have been widely used and validated from previous studies for buckling [8,9], injury/ trauma [20], and falls [21].

The questions about knee buckling and injury for both control period and case period questionnaires were the same:

1) Did you have an injury to your index knee during the last 7 days that limited your activities? How did this injury occur?

2) Did you meet with an incident in the past 2 days where your knee buckled or gave way? How many times in the past 2 days did you experience such an episode? As a result of knee buckling or giving way, did you accidentally fall and hit the floor or ground? In general what were you doing when your knee buckled?

### Statistical Analysis

Descriptive statistics were computed for all participants as well as for those with at least one case period and one control period. Comparisons of the baseline characteristics between participants with both case (exacerbation) and control periods and those without discordant outcomes were assessed using a chi-square test for categorical variables and analysis of variance for continuous variables when normality and homogeneity of variance assumptions were satisfied.

As each participant could contribute multiple case and control periods, an m:n matched study design was used to assess the relation of knee buckling and knee injury to risk of knee pain exacerbation using conditional logistic regression (SAS PHREG, v. 9.4). Only participants with both case and control periods were used in the regression analysis as participants with only case or only control periods will not contribute information for such an analysis (ie, no information for comparing differences in presence of the study triggers between exacerbation and control periods within an individual). To ensure a no overlap between any analyzed periods, only questionnaires with at least a 7-day interval between any two assessments were used.

## Results

Among 297 subjects recruited into the study, 157 had data on both case and control periods (Table 1). Out of 795 Web contacts where the participants said that they were experiencing an increase in pain, only 571 (71.8%) were identified by the system as having a pain exacerbation (case period) based on the required criteria (an increase of ≥ 2 on a 0-10 numerical rating scale). Out of 571 case periods reported, 513 (89.8%) were reported at one of their regular control period visits.

**Table 1:** Demographic characteristics of study participants.

	Study participants with both case and control periods^a ^(n=157)	Participants with only case or control periods but not both (n=140)	*P* value
Female, n (%)	103 (65.6)	79 (56.4)	.12^b^
Age (year), mean (SD)	61.8 (8.4)	62.4 (7.7)	.55^c^
BMI^d^(kg/m^2^), mean (SD)	29.5 (5.6)	29.1 (5.1)	.48^c^
KOOS^e^Symptoms mean(SD)	44.2 (14.1)	44.8 (11.7)	.72^c^
KOOS Pain, mean (SD)	55.1 (15.9)	56.1 (17.4)	.59^c^
KOOS ADL^f^, mean (SD)	62.0 (19.7)	63.3 (19.0)	.57^c^
KOOS Sport/Recreation mean(SD)	22.3 (23.8)	22.4 (22.7)	.96^c^
KOOS QOL^g^, mean (SD)	39.6 (17.7)	41.7 (18.8)	.33^c^

^a^The study participants included in the case-crossover analysis; participants with only case or control periods are in the right column and did not contribute information for the conditional logistic regression analyses.

^b^chi-square test

^c^ANOVA test

^d^BMI: body mass index

^e^KOOS: knee injury and osteoarthritis outcome score

^f^ADL: function in daily living

^g^QOL: quality of life

During the 3-month follow-up period, 400 knee pain exacerbations occurred. Among the participants, no statistically significant differences were seen in the baseline characteristics between those who had both case and control periods and those who did not; although there was a greater proportion of women among those who had both periods, which may be clinically relevant (Table 1).

There were 102 out of 1244 (8.2%) reports of injury to the index knee during the previous 7 days and 249 (19.9%) reports of knee buckling or giving way in the previous 2 days. The most frequent causes of injury were sport injuries (20.0%), tripping (13.0%), slipping (11.0%), and falling (11.0%). Of the 297 participants, 131 (44.1%) participants complained of one or more buckling episodes during the 3 months of follow up. The most common activity reported when buckling occurred was walking on level ground (52.9%), followed by twisting or turning (18.8%), and going upstairs or downstairs (17.8%). 71 participants (54.2%, 71/131) reported that buckling occurred two to five times, 47 participants (35.9%, 47/131) reported that the buckling occurred only once, and 17 (13.0%, 17/131) participants reported a fall during buckling.

About 81.6% (83 out of 102) of participants who reported a knee injury and 85.5% (213 out of 249)who reported knee buckling indicated that their pain exacerbation started within the last 2 days.

As shown in Table 2, the odds ratio of experiencing a pain exacerbation with an injury in the previous 7 days was 10.2 (95% CI 5.4, 19.3). For an event of knee buckling in the previous 2 days the odds ratio of experiencing a pain exacerbation was 4.0 (95% CI 2.6, 6.2). Furthermore, there was a dose–response relationship between the number of knee buckling episodes and the risk of knee pain exacerbation. The odds ratios of knee pain exacerbation were 3.5 (95% CI 2.0, 6.0), 4.1 (95% CI 2.4, 7.0), and 20.1 (95% CI 3.7, 110), respectively, if participants experienced knee buckling once, 2-5 times, and more than 5 times during the previous 2 days (Table 3).

**Table 2:** Association of knee injury and risk of knee pain exacerbation.

Knee injury	Case periods (N)	Control periods (N)	Odds ratio (95% CI)
No	329	820	1.0 (referent)
Yes	71	31	10.2 (5.4, 19.3)

**Table 3:** Association of knee buckling and risk of knee pain exacerbation.

		Case periods (N)	Control periods (N)	Odds ratio (95% CI)
Knee buckling	No	259	743	1.0 (referent)
	Yes	141	108	4.0 (2.59, 6.18)
Number of episodes	1	64	54	3.5 (2.0, 6.0)
	2-5	66	50	4.1 (2.4, 7.0)
	≥ 6	11	4	20.1 (3.7, 110)

## Discussion

This study demonstrates a strong association between knee buckling and injury with pain exacerbation in people with knee OA. Of these mechanical insults, knee injury provides the most potent risk of exacerbation; albeit injury occurred less frequently in our cohort than buckling.

The most common type of knee injury was sustained as a consequence of sports injuries and tripping. Of the participants who complained of buckling, the most common frequency was from two to five times, whereas more than six buckling episodes were associated with the highest odds ratio suggesting some dose response effect. Knee buckling may be associated with an increased risk of falling and furthermore with a risk of fractures [8], however in our study only a small percentage (13%, 17 out of 131) of participants fell during an event of buckling. When buckling occurred, walking on level ground was by far the most common activity preceding an episode of buckling.

An injury to the synovial joint may lead to an exacerbation of pain by leading to an inflammatory response and release of chemical mediators into the joint [7]. Furthermore, primary afferent nerves may be sensitized and otherwise harmless movements may become more painful [7].

### Principal Findings

Our findings are consistent with the hypothesis that knee injuries and buckling are important trigger factors for knee pain exacerbation. By implication, it is possible that preventing knee buckling and knee injuries can reduce pain exacerbation. Instability of the knee, a main cause of knee buckling is associated with quadriceps weakness and is thought to be highly treatable [22]. Quadriceps strengthening and balance training are elements of successful rehabilitative efforts to treat knee instability [22,23]. It can be speculated that this type of training might prevent knee buckling, and therefore, reduce the reoccurrence of pain exacerbation episodes.

As many injuries result from slipping or tripping, modifying falls risk or the predisposition to such, could assist in the prevention of knee injury. Cognizant of the fact that our study sample is younger than those typically at greater risk of falls, one potentially important piece of advice for elderly patients is to wear proper footwear indoors, as many falls occur due to lack of fixation in the shoe or due to an excessively soft sole [24,25]. A person is likely to slip or trip due to improper shoes sizes (either too small or too large)—a common risk factor for falls [25].

Neuromuscular exercise training is beneficial in prevention of knee injury and is known to be a promising avenue in reducing knee injury risk [26]. It aims to improve control and alignment of the knee during functional activities [27]. Neuromuscular exercise and strengthening exercise were compared with each other and it was found that both similarly improved pain and function [28].

### Limitations

Possible limitations of our study include incomplete data in questionnaires, in addition to some potential for recall bias and participant fatigue. Every participant was followed regularly for 3 months and the possibility that they may have logged-on during a pain exacerbation is assumed to be null. There is potential for recall bias given that a participant is first asked questions about a recent knee pain exacerbation, which is followed by questions asking them to recall knee-related events that occurred during the same recall interval. The potential effects of time-varying potential predictors including medication, shoe wear, and weather have not been controlled in these analyses. Similarly, after accounting for potential overlap, the independent effects of buckling and injury have not been assessed.

For defining knee injury, we included injuries occurring in daily life without further delineation, for example, falling over, slipping, tripping, twisting the ankle, or landing from a jump. This may lead to misclassification that could reduce the effects found. Furthermore, if a person experiencing a pain exacerbation wonders why this may have occurred, an open-ended definition of the exposure invites an opportunity for recall bias.

Of the participants, 82% (83 out of 102) who reported that they had a knee injury during the last 7 days indicated that their pain exacerbation started within the last 2 days, whereas, 86% (213 out of 249) of those participants who reported knee buckling indicated that their pain exacerbation started within the last 2 days. All pain exacerbations either overlap the buckling recall period or precede it. We have assumed that the knee injury and/or buckling may play a triggering role for pain exacerbation but causation cannot be proven in this study and reverse causality remains a possibility. It is entirely plausible for pain exacerbations to be a “trigger” for increased knee instability and falls, both of which could themselves be a source of a knee injury.

Psychosocial aspects contribute to the genesis of pain, as people with lower self-efficacy or higher catastrophizing have a higher perception of pain [29]. Patients often develop anxiety about pain exacerbations, which can lead to avoidance of activities, depression, or sleep-onset insomnia [5,30]. As each participant acts as his or her own control, constant personal factors are controlled via the case-crossover study design and cannot bias our results. Catastrophizing and self-efficacy are considered traits that should not change unless intervened upon and also not likely change over a 3-month period. On the other hand, mood fluctuations, for instance, could certainly influence these results and will be examined in future analyses. We have also collected data on other time-varying potential predictors including medication, shoe wear, and weather, which will also be examined in future analyses.

### Conclusions

This investigation demonstrated the potential importance of knee injury and buckling as triggers for knee pain exacerbation in participants with symptomatic knee OA. These findings make a noteworthy contribution to the etiology of knee pain in OA and potentially counseling of persons with knee OA to avoid activities that lead to knee injuries or buckling.

## Abbreviations

KLG: Kellgren and Lawrence Grade
NRS: Numerical rating scale
OA: Osteoarthritis
